# Central Feminization of Obese Male Mice Reduces Metabolic Syndrome

**DOI:** 10.3390/brainsci12101324

**Published:** 2022-09-30

**Authors:** Katherine Blackmore, Colin N. Young

**Affiliations:** School of Medicine and Health Sciences, George Washington University, 2300 I Street NW, Washington, DC 20037, USA

**Keywords:** obesity, adipose, fatty liver, neural control, estrogen

## Abstract

Metabolic syndrome encompasses a spectrum of conditions that increases the risk for cardiovascular and metabolic diseases. It is widely accepted that the sex hormone estrogen plays a protective metabolic role in premenopausal women, in part through central nervous system (CNS) mechanisms. However, most work to date has focused on the loss of estrogen in females (e.g., menopause). Interestingly, transgender individuals receiving feminizing gender affirming therapy (i.e., estrogen) are relatively protected from metabolic syndrome conditions, pointing to a role for CNS estrogen in the development of metabolic syndrome in men. Here, we show that estrogen signaling in the brain protects males from metabolic syndrome and obesity related complications. First, short-term CNS specific supplementation of low-dose 17-β-estradiol in diet-induced obese male mice resulted in a significant reduction in body weight in parallel with a decrease in food intake without alterations in energy expenditure. In conjunction, central supplementation of estrogen reduced visceral adiposity, including epididymal and abdominal regions, with slighter decreases in subcutaneous inguinal and thermogenic brown adipose tissue. Furthermore, central estrogen administration reduced the liver manifestation of metabolic syndrome including hepatomegaly and hepatic steatosis. Collectively, these findings indicate that a lack of estrogen action in the brain may predispose males to metabolic syndrome pathogenesis.

## 1. Introduction

Metabolic syndrome, also known also as Syndrome X, is defined by the World Health Organization as a pathologic condition characterized by a constellation of metabolic abnormalities including increased body weight, abdominal obesity, and hyperlipidemia [1,2,3]. Individuals with metabolic syndrome are at an increased risk for a variety of conditions, such as type II diabetes, cardiovascular disease, and fatty liver [4]. While the etiology of metabolic syndrome is multifactorial, sexual dimorphism is clearly established. Adult men display increased prevalence of metabolic abnormalities when compared to fertile women [5,6]. Interestingly, post menopause, the incidence of metabolic syndrome in women is significantly increased [7,8]. It is widely proposed that the increased post-menopausal incidence is due in part to estrogen deficiency caused by progressive ovarian senescence [7,8,9]. Additionally, post-menopausal women that receive hormone replacement therapy or oral contraceptives have a lower incidence of metabolic syndrome when compared to age matched controls [10,11]. Collectively, this can be interpreted as indirect evidence that endogenous estrogen plays a metabolic protective role in females.

Classically, studies have relied on the removal of estrogen, through ovariectomy or estrogen antagonism, or the removal of testosterone through gonadectomy to study the sexual dimorphism of metabolic abnormalities. However, these methods oversimplify the role of sex hormones in metabolic homeostasis as they often investigate the dominant sex hormone with little evaluation of alternate sex hormone signaling (e.g., either testosterone in women or estrogen in men). Interestingly, transgender individuals receiving feminizing gender affirming therapy (i.e., estrogen) appear to be relatively protected from metabolic syndrome conditions, including type II diabetes, insulin resistance, and cardiovascular dysfunction [12,13]. However, global estrogen replacement poses numerous hurdles. In particular, estrogen has tissue specific physiological effects, both positive and negative [14,15,16]. Thus, identification of specific sites of estrogen action is imperative to develop selective estrogen therapies that can treat diseases associated with obesity.

It is well accepted that estrogen signaling in the central nervous system (CNS) is critically involved in metabolic regulation including consummatory behavior, glucose homeostasis, body weight control, and hepatic regulation [17,18,19,20]. Interestingly, in males, whole body removal of estrogen receptor-α or knockout of the receptor selectively in the brain results in metabolic syndrome phenotypes including increased body weight and abdominal obesity [21,22]. Moreover, knockdown of estrogen receptor-α selectively in the ventromedial hypothalamus in males results in increased body weight [23], although further studies delineating brain nuclei involved are need given contrasting findings [22]. Nevertheless, these findings indicate that endogenous estrogen signaling may partially protect males from metabolic syndrome and furthermore, that supplementation of estrogen may provide additional protective effects against obesity-related diseases. With this in mind, we used a unique model of central feminization of obese male mice to test the hypothesis that CNS estrogen signaling is necessary to protect males against metabolic syndrome complications.

## 2. Materials and Methods

### 2.1. Experimental Model

Male C57BL/6J mice (strain#: 000664) were obtained from Jackson Laboratory and housed on a 12 h light–dark cycle with ad libitum access to food and water. Mice were singly housed and fed a high fat diet (HFD; 60% kcal from fat; Research Diets Inc., New Brunswick, NJ, USA) for a period of 10 weeks starting at 6 weeks of age. All experimental procedures were approved by the Institutional Animal Care and Use Committee at the George Washington University and met the standard guidelines set forth by the National Institutes of Health Guide for the Care and Use of Laboratory Animals.

### 2.2. Pharmacological Agents

The steroid hormone 17-β-estradiol (Sigma-Aldrich, St. Louis, MO, USA) was dissolved in sterile saline to the desired concentration (1.5 μg in 1 μL). This dose of 17-β-estradiol has been previously confirmed to mimic circulating concentrations of estrogen in female mice during the pro-estrous phase [24,25,26].

### 2.3. Lateral Cerebroventricular Cannulation

Following 10 weeks of HFD, mice were anaesthetized with ketamine (100 mg kg^−^^1^) mixed with xylazine (10 mg kg^−^^1^). Mice were secured in a stereotaxic device and the surface of the skull was visualized with a dissecting microscope. An intracerebroventricular (ICV) cannula was implanted into the left ventricle (PlasticsOne Inc., Roanoke, VA, USA) for injection of 17-β-estradiol or vehicle control (sterile saline) using the following coordinates from *Bregma*: 0.3 mm rostral, 1.0 mm, and 3.2 mm ventral from the dorsal surface of the skull. Two anchor screws were placed posterior to the cannula, and dental cement was used to fix the cannula and screws in place. Subcutaneous injection of ketofen (2.5 mg kg^−^^1^) was provided prior to surgery and 24 h post-surgery for pain management. Mice were given 7 days of recovery prior to ICV administration of 17-β-estradiol or vehicle control once per day over 6 days [24,27,28].

### 2.4. Indirect Calorimetry

Body weight, food/water intake, energy expenditure, oxygen consumption, and respiratory quotient were recorded using a Promethion metabolic measurement system (Sable Systems, Las Vegas, NV, USA). Measures were obtained for 72 h prior and continuously during daily ICV 17-β-estradiol or saline supplementation. Data were analyzed using custom macros in ExpeData software (Sable Systems).

### 2.5. Liver Histology

Neutral lipids were stained using a standard Oil Red O protocol as previously described [29]. Fresh liver tissue was embedded in optimal cutting temperature fixation medium and stored at −80 °C. Frozen liver tissue was cryosectioned at 14 μm and equilibrated in 60% isopropyl alcohol. Liver sections were then incubated in fresh, filtered Oil Red O solution (Alfa Aesar, Haverhill, MA, USA) for 7 min, washed in 60% isopropyl alcohol, and briefly rinsed in running tap water. A water-soluble mounting media (Vectashield HardSet, Vector Laboratories, Newark, CA, USA) was used for mounting and sections were imaged using light microscopy (Olympus BX43; Olympus, Center Valley, PA, USA). Tissue lipid accumulation was quantified by area fraction analysis using ImageJ software (National Institute of Health) in 18 sections from each animal and averaged to calculate the level of steatosis.

### 2.6. Quantification and Statistical Analysis

Data are expressed as mean ± SEM. Students two-tailed unpaired *t* tests were used for comparisons between two groups. Group and temporal analysis were performed using a two-way repeated measures ANOVA. Significance was set at *p* < 0.05.

## 3. Results

### 3.1. Central Feminization of Obese Male Mice Reduces Body Weight and Food Intake

**Figure 1:**
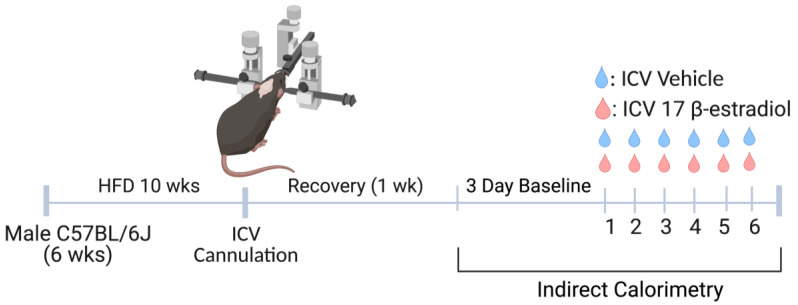
**Schematic overview of the experimental protocol.** Male C57Bl/6J mice were fed a high fat diet (HFD) for 10 weeks and then instrumented with an intracerebroventricular (ICV) cannula. Following recovery and baseline indirect calorimetry measurements, ICV 17-β-estradiol (1.5 μg in 1 μL) or vehicle control (saline) was administered once daily over 6 days.

Building upon evidence that estrogen replacement therapy in transgender females is protective against metabolic syndrome [12,13], we aimed to investigate whether central estrogen supplementation in males would influence metabolic syndrome phenotypes. To evaluate this, obese male mice were instrumented with ICV cannulas and received daily 6-day administration [24,28] of low-dose central 17-β-estradiol administration (Figure 1).

**Figure 2:**
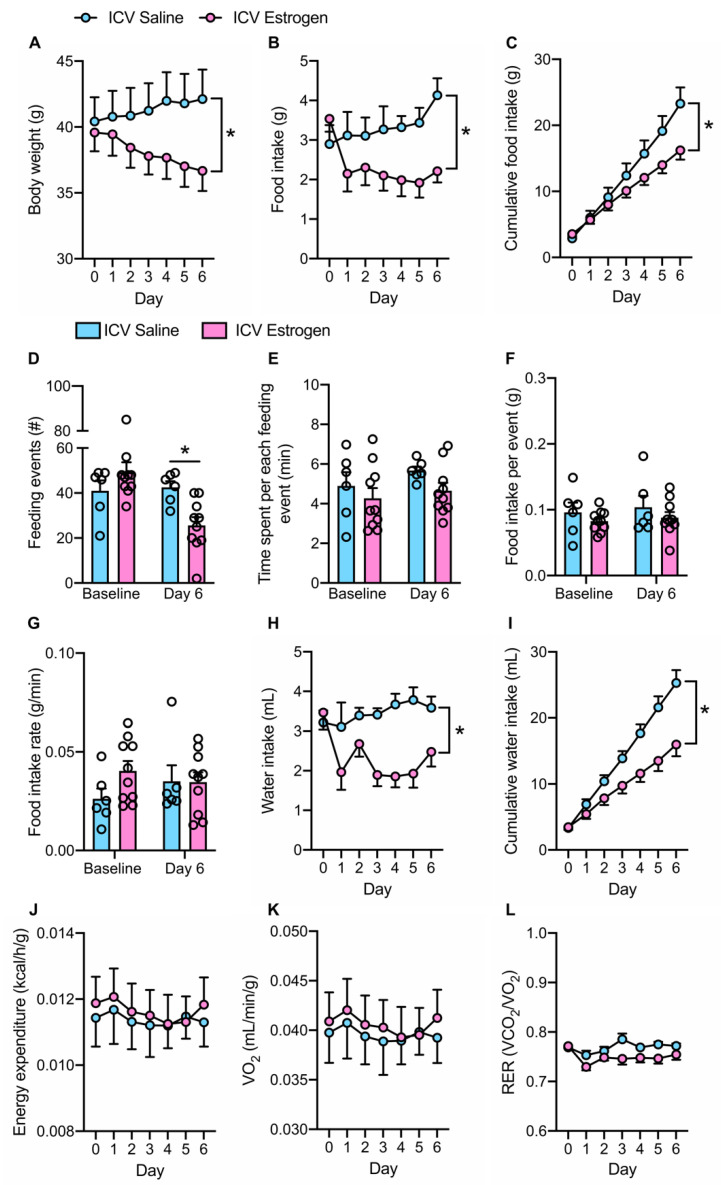
**Central estrogen supplementation decreases body weight, food intake and water intake in obese males.** (**A**) Body weight, (**B**) daily food intake, and (**C**) cumulative food intake at baseline and during 6-day administration of ICV 17-β-estradiol or saline control in obese male mice. (**D**) Feeding events, (**E**) time spent per each feeding event, (**F**) food consumed per feeding event, and (**G**) food intake rate at baseline and following 6-day administration of ICV 17-β-estradiol or saline control. (**H**) Daily water intake, (**I**) cumulative water intake, (**J**) energy expenditure, (**K**) oxygen consumption, and (**L**) respiratory exchange ratio at baseline and during 6-day administration of ICV 17-β-estradiol or saline control in obese male mice. n = 8–11. * *p* < 0.05 vs. ICV Saline.

Central estrogen supplementation resulted in a progressive loss in body weight in contrast to saline controls which tended to gain weight (Figure 2A). In parallel, central estrogen resulted in a reduction in food intake, which was evident when quantified as both daily food intake (Figure 2B) and cumulative food intake across the study period (Figure 2C). This reduction in caloric intake appeared to be primarily due to a reduction in the number of feeding events (“meals”) across a 24-h period (Figure 2D), whereas the amount of time spent, food consumed, and rate of food intake per meal remained similar between ICV estrogen and saline groups (Figure 2E–G). Consistent with the documented influence of brain estrogen signaling on fluid balance [30], daily (Figure 2H) and cumulative (Figure 2I) water intake were also reduced in response to central estrogen supplementation.

Interestingly, central estrogen-related reductions in body weight and food/water intake occurred without significant changes in energy expenditure or oxygen consumption when compared to saline controls (Figure 2J,K). Additionally, using respiratory exchange ratio as an indicator of substrate utilization also pointed to no differences between groups (Figure 2L). Together, these findings indicate that central estrogen supplementation in obese male mice results in a loss in body weight that is likely mediated by a reduction in food intake.

### 3.2. Central Estrogen Supplementation Reduces Visceral Adiposity and Rescues Hepatic Steatosis

**Figure 3:**
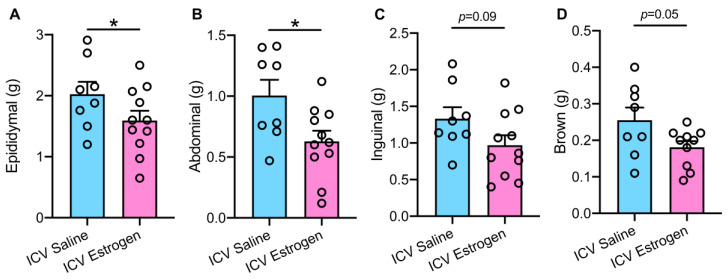
**ICV supplementation of estrogen in males selectively reduces visceral adiposity during obesity.** (**A**) Visceral epididymal, (**B**) visceral abdominal, (**C**) subcutaneous inguinal, and (**D**) thermogenic brown adipose mass following 6-day administration of ICV 17-β-estradiol or saline control in obese male mice. n = 8–11. * *p* < 0.05 vs. ICV Saline.

Considering that short-term central estrogen administration reduced body weight in obese male mice, we next evaluated several regional adipose depots. A significant reduction in visceral white adipose tissue masses, including epididymal (Figure 3A) and abdominal (Figure 3B) regions was noted in ICV estrogen treated animals when compared to saline controls. Similarly, slight reductions in subcutaneous inguinal (Figure 3C) and thermogenic brown (Figure 3D) adipose tissue were seen, although the decrease in these adipose depots did not appear as robust as the decline in white adiposity.

**Figure 4:**
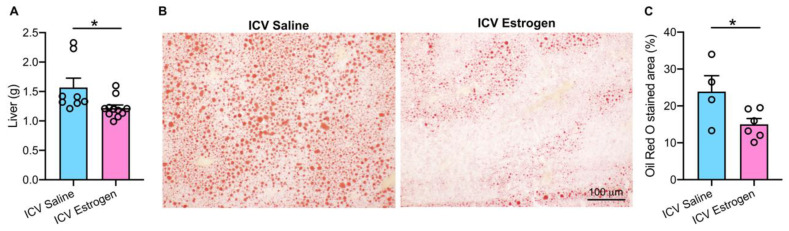
**Central supplementation of estrogen in obese male mice alleviates hepatomegaly and hepatic steatosis.** (**A**) Liver weight (n = 8–11), (**B**) representative hepatic Oil-Red-O staining, and (**C**) quantitative analysis of Oil-Red-O-stained area (n = 4–6) following 6-day administration of ICV 17-β-estradiol or saline control in obese male mice. * *p* < 0.05 vs. ICV Saline.

We subsequently turned our attention to the liver given that the hepatic manifestation of metabolic syndrome is non-alcoholic fatty liver disease characterized by steatosis [31,32,33]. Acute central estrogen supplementation resulted in a clear reduction in hepatomegaly (Figure 4A) in HFD fed males. Histological evaluations using Oil Red O staining of liver neutral lipids further revealed a reduction in hepatic lipid vesicle accumulation following short-term ICV estrogen administration (Figure 4B). This was confirmed with quantitative analysis where a marked ~40% reduction in hepatic steatosis occurred in obese males treated with central estrogen over 6 days (Figure 4C). Collectively, these findings indicate that central supplementation of estrogen in obese male mice reduces adiposity and rescues hepatic steatosis.

## 4. Discussion

We report here a unique role for estrogen supplementation in the male brain. First, our data indicate that central supplementation of 17-β-estradiol in obese male mice results in a significant reduction in body weight and food intake. This is accompanied by a clear loss of visceral adiposity with subtle reductions in inguinal and thermogenic brown adipose depots. Moreover, we provide evidence that central estrogen administration reduces the liver manifestation of metabolic syndrome including hepatomegaly and hepatic steatosis. Collectively, these findings indicate that a lack of estrogen signaling in the brain may predispose males to the pathogenesis of metabolic syndrome.

Evidence from both humans and laboratory animals suggests that estrogen plays an important role in body weight regulation [24,34]. In humans, meta-analyses of approximately one-million women suggested a positive correlation between progressive ovarian senescence (decreased circulating estrogen) and increased weight gain [35,36]. Similarly, ovariectomy of female mice causes a significant increase in body weight when compared to sham controls. This is easily alleviated following estrogen replacement [37]. Within the brain, earlier studies indicated that CNS specific estrogen receptor knockout also results in increased body weight gain in both males and females [20,22,38]. Our work extends these conclusions by confirming that brain-specific estrogen replacement ameliorates body weight gain during HFD feeding in males. Interestingly, in both humans and rodents, body weight gain following estrogen reduction is not accompanied by changes in energy expenditure [17,21]. In line with this available literature, central supplementation of estrogen in obese male mice did not significantly alter energy expenditure, suggesting a role for another physiological mechanism, such as food intake, in estrogenic body weight regulation.

In rodents, the release of estradiol from the ovaries occurs cyclically during a 4–5-day estrous cycle. Apart from changes in sexual behavior, the estrous cycle causes fluctuations in food intake in rhythm with circulating estrogen concentrations. For example, food intake is drastically reduced during the evening of pro-estrous, following the rise of circulating estrogen [25,39,40]. This has been further confirmed in humans showing a 350–600 kcal food reduction during the ovulatory phase, when estrogen concentrations are highest [41]. Conversely, ovariectomy in female rodents or non-human primates results in hyperphagia [24,40]. Building upon this evidence, the current findings indicate that central estrogen supplementation in obese males reduces caloric intake, primarily through a decrease in the number of feeding events per day. Importantly, this was accompanied by a reduction in body weight. While we cannot exclude an indirect effect of estrogen on modulating testosterone levels [42,43], these findings suggest that a decrease in energy intake may be a key driver by which CNS estrogen administration reduces body mass in males.

Body fat is traditionally distributed into two broad compartments, subcutaneous/thermogenic and visceral adipose tissue, which are differentiated by distinct biochemical and metabolic features. Interestingly, during obesity, men and women in general store adipose tissue in different depots. It is commonly accepted that men are more prone to visceral adiposity whereas women tend to store fat in subcutaneous regions [44,45]. Interestingly, visceral fat varies inversely with estrogen levels. Low circulating estrogen, either through menopause or genetic variation (such as Kleinfelder’s disease), causes a significant increase in visceral fat accumulation [46]. Additionally, ovariectomized female mice quickly gain visceral fat mass with no change in subcutaneous depots [37]. While peripheral estrogen action is clearly involved in adipose metabolism [21,37], existing evidence also points to a key role for central estrogen signaling in the regulation of regional adipose distribution. For example, knockdown of estrogen receptor-α in the ventromedial hypothalamus of female mice promotes visceral fat deposition [20]. Here, we extend this work by demonstrating what we believe is the first report of a critical role for exogenous estrogen supplementation in body fat distribution *in males*. Specifically, central administration of estrogen significantly reduced visceral adipose tissue in obese male mice. This is of particular clinical relevance as abdominal/visceral obesity, where adipose surrounds the intraabdominal organs, is directly linked to numerous pathological conditions including impaired glucose and lipid metabolism, insulin resistance [47,48,49], and obesity related mortality [50,51]. Interestingly, central supplementation of estrogen also resulted in slight, albeit non-significant, reductions in subcutaneous and brown adipose tissue in obese male mice, warranting further exploration on estrogenic control of adipose distribution. Collectively, these findings indicate that a lack of brain estrogen signaling in men may predispose to the development of visceral adiposity, and supplementation of estrogen may be a unique strategy to selectively target visceral adiposity while partially sparing beige/brown adipose.

Non-alcoholic fatty liver disease, considered the hepatic manifestation of metabolic syndrome, encompasses a spectrum of steatogenic liver diseases that is associated with increased type II diabetes [52] and obesity related mortality [53]. Peripheral estrogen signaling is intricately linked to hepatic metabolic abnormalities in males. For example, aromatase knockout male mice, which lack the ability to produce endogenous estrogen, develop hepatic steatosis that can be reversed through estrogen replacement [54]. Although rare, several cases of human aromatase deficiency have also been reported in the literature. Men with mutations in the Cyp19 gene (which encodes aromatase) show lipid profiles similar to patients with metabolic syndrome including elevated triglyceride levels and low concentrations of high-density lipoproteins, indicative of fatty liver disease [55,56,57]. A similar metabolic phenotype is seen in male mice lacking functional estrogen receptors. Following global estrogen receptor knockout, male mice display abdominal obesity and increased hepatic triglycerides [21]. The current data corroborate that estrogen in obese males protects against hepatic steatosis and further points to the brain as a critical site of action. While the specific mechanism through which CNS estrogen reduces fatty liver in males remains open for investigation, it is possible that estrogen mediated reductions in hepatic steatosis occurs through direct or indirect mechanisms. A reduction in food intake could shift fuel utilization to hepatic lipids, thus reducing hepatic steatosis indirectly. However, the lack of change in respiratory exchange ratio toward lipid utilization in the current dataset would argue against this. Alternatively, it is plausible that central estrogen in males directly influences hepatic metabolism via autonomic and/or endocrine networks given emerging literature pointing to a key role for the brain in the modulation of liver metabolism [33,58,59]. However, we cannot exclude the fact that central estrogen supplementation to males may alter circulating estrogen and/or testosterone levels [42,43] although this is unlikely given previous findings using the dose that was applied in the current studies [28]. Moreover, we acknowledge that estrogen supplementation may reduce testosterone levels, although findings in this regard have been equivocal [59,60,61]. Whether brain specific estrogen supplementation in males reduces central testosterone concentrations is yet to be determined. Nevertheless, the current findings highlight a novel role for CNS estrogen to reduce obesity-related hepatic steatosis in males, although further work is needed.

In summary, the current findings reveal a novel role for central estrogen administration in obese male mice. Notably, central supplementation of 17-β-estradiol reduces body weight, food intake, and adiposity, without changing energy expenditure in obese male mice. Additionally, central estrogen dosing ameliorates hepatomegaly and hepatic steatosis. Collectively, these studies point to a potential protective and therapeutic role for estrogen in the male brain during obesity. Moreover, these data may help explain why premenopausal females and transgender females are relatively protected from metabolic syndrome.

## Data Availability

The data presented in this manuscript are available upon request from the corresponding author.
